# Individual and Combined Associations of Glucose Metabolic Components With Cognitive Function Modified by Obesity

**DOI:** 10.3389/fendo.2021.769120

**Published:** 2021-12-13

**Authors:** Ruixin He, Ruizhi Zheng, Jie Li, Qiuyu Cao, Tianzhichao Hou, Zhiyun Zhao, Min Xu, Yuhong Chen, Jieli Lu, Tiange Wang, Yu Xu, Yufang Bi, Weiqing Wang, Mian Li, Yan Liu, Guang Ning

**Affiliations:** ^1^ Department of Endocrine and Metabolic Diseases, Shanghai Institute of Endocrine and Metabolic Diseases, Ruijin Hospital, Shanghai Jiaotong University School of Medicine, Shanghai, China; ^2^ Shanghai National Clinical Research Center for Metabolic Diseases, Key Laboratory for Endocrine and Metabolic Diseases of the National Health Commission of the PR China, Shanghai National Center for Translational Medicine, Ruijin Hospital, Shanghai Jiaotong University School of Medicine, Shanghai, China; ^3^ Department of Endocrinology, The Third People’s Hospital of Datong, Datong, China

**Keywords:** glucose metabolism, insulin resistance, glycemic level, cognitive function, obesity, metformin

## Abstract

**Aim:**

We aimed to detect the individual and combined effect of glucose metabolic components on cognitive function in particular domains among older adults.

**Methods:**

Data of 2,925 adults aged over 60 years from the 2011 to 2014 National Health and Nutrition Examination Survey were analyzed. Individuals’ cognitive function was evaluated using the Digit Symbol Substitution Test (DSST), the Animal Fluency Test (AF), the Consortium to Establish a Registry for Alzheimer’s Disease Immediate Recall (CERAD-IR), and CERAD Delayed Recall (CERAD-DR). Participants’ glucose metabolic health status was determined based on fasting plasma glucose, insulin, homeostasis model assessment of insulin resistance (HOMA-IR), glycated hemoglobin (HbA_1c_), and 2-h postload glucose. Linear regression models were used to delineate the associations of cognitive function with individual glucose metabolic component and with metformin use. Logistic regression models were performed to evaluate the associations of cognition with the number of glucose metabolic risk components.

**Results:**

CERAD-IR was significantly associated with HOMA-IR and insulin. HbA_1c_ was related to all the cognitive tests except AF. Among participants without obesity, HOMA-IR and insulin were both negatively associated with CERAD-IR and CERAD-DR. Odds of scoring low in DSST increased with the number of glucose metabolic risk components (odds ratio 1.94, 95% confidence interval [CI] 1.26 to 2.98). Metformin use was associated with better performance in DSST among diabetes patients (*β* = 4.184, 95% CI 1.655 to 6.713).

**Conclusions:**

Our findings support the associations of insulin resistance and glycemic level with cognitive function in key domains, especially among adults without obesity. There is a positive association between metformin use and cognition.

## Introduction

The prevalence of type 2 diabetes has increased remarkably over the past decades (1), imposing huge burden on healthcare expenditure. Dementia has witnessed a similar population trend. The number of people living with dementia worldwide in 2015 was estimated at 47.47 million, and reaching 135.46 million by 2050 (2). Previous literatures demonstrated increased odds of cognitive impairment in relation to diabetes (3); the former, in turn, results in worse diabetes management, more frequent occurrence of severe hypoglycemic episodes, an increased risk of cardiovascular events, and death (4). Therefore, comprehensive understanding and management of glucose metabolic risk factors have become an imperative issue for the prevention of cognitive dysfunction.

With respect to risk factors for cognitive impairment among patients with diabetes, glucose metabolic components, including glycemic level and insulin resistance, have attracted much attention. Some studies have shown that high glycated hemoglobin (HbA_1c_) was detrimentally related to cognitive functions (5), whereas limited research has focused on the associations of cognition with other glucose metabolic indicators, such as fasting plasma glucose (FPG) and 2-h postload glucose (2h-PG). Regarding disorders of insulin homeostasis, converging evidence has shown that insulin resistance or consequent hyperinsulinemia is related to poor cognitive performance (6). However, most of these studies focused on the relationship of global cognitive function with glycemic level or insulin homeostasis separately, and few have systematically described individual and combined associations of glucose metabolic components with cognition in particular domains, such as learning, memory, processing speed, executive function, and language.

Additionally, given the relationship of obesity with both diabetes (7) and neurodegenerative diseases (8), it is of substantial interest to investigate whether any potential association of glucose metabolic components with domain-specific cognitive functions could be modified by obesity status. Furthermore, since diabetes medications, especially metformin, have been implicated to be associated with slowed rate of cognitive decline by previous studies (9, 10), we also tested this hypothesis in the current study population.

Several studies have investigated associations between diabetes and cognitive function using data from earlier National Health and Nutrition Examination Survey (NHANES), which lacked information on cognitive function in key domains measured comprehensively among older adults. Two studies based on 1999–2002 NHANES (11, 12) assessed the association of FPG, insulin resistance, and metabolic syndrome with cognition, but only implemented Digit Symbol Substitution Test (DSST) to evaluate cognitive function. A 1988–1994 NHANES study (13) investigated the associations between diabetes combined with hypertension and cognitive function, but the study population was restricted to individuals aged between 20 and 59, so the external validity for elder populations is uncertain. Another study (14) using NHANES 2011–2014 cycles, found lower DSST and Animal Fluency Test (AF) scores in relation to higher HbA_1c._ However, evidence regarding the relationship of cognitive dysfunction with a broad spectrum of glucose metabolic components, such as insulin resistance, obesity status, and metformin usage, is still limited.

Therefore, taking advantage of NHANES, a nationally representative sample of US older adults, we aimed to determine the individual and combined associations of glucose metabolic components with cognitive functions in key domains, and whether such associations were modified by obesity status. We also test the hypothesis that metformin use is associated with better cognitive function in elder adults with diabetes.

## Materials and Methods

### Study Population

**Figure 1:**
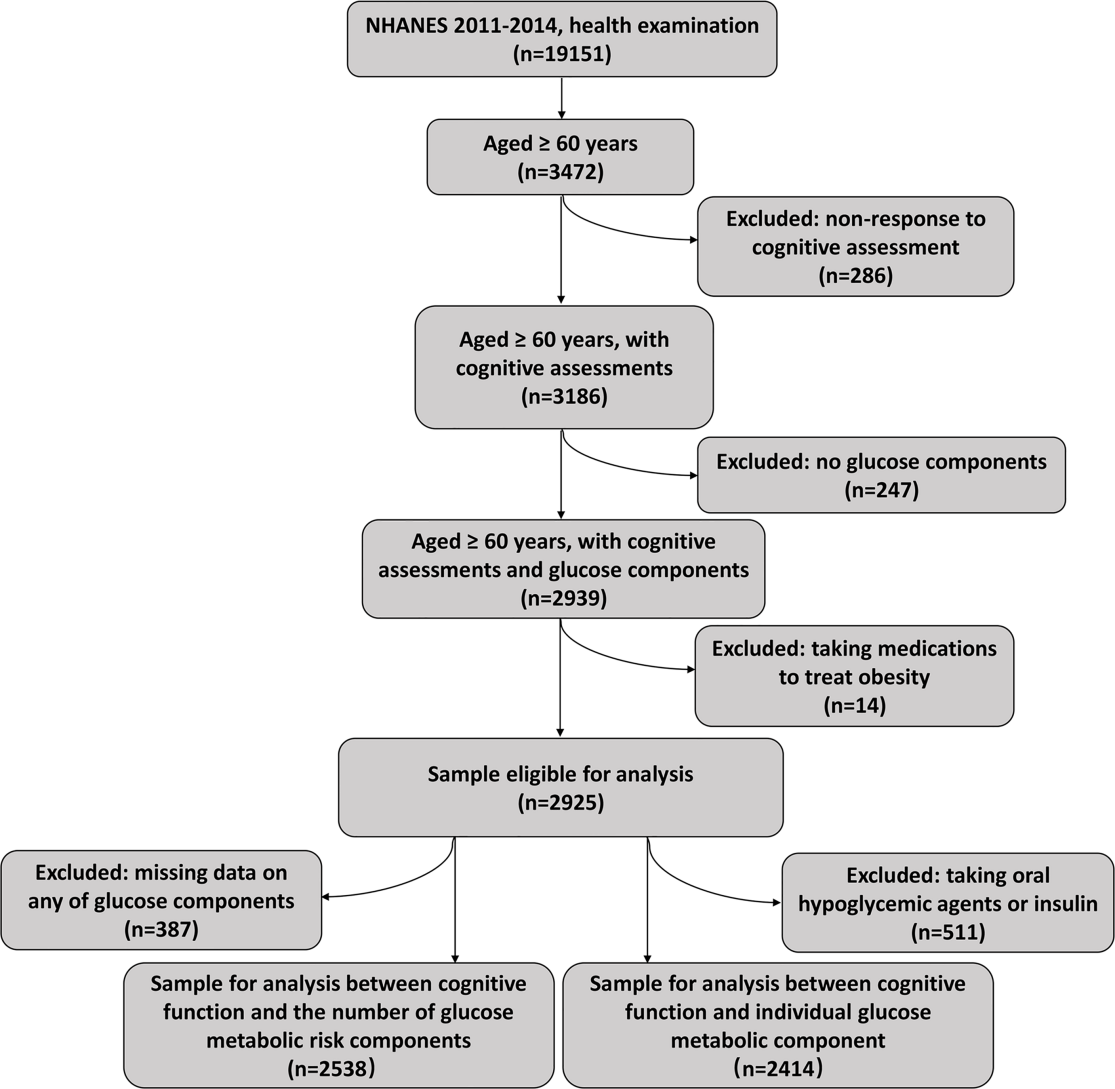
Flow chart showing pipeline of selecting subjects using NHANES 2011–2014. NHANES, National Health and Nutrition Examination Survey.

NHANES is a nationwide continuous cross-sectional survey that assessed nutritional and health status of civilians in the United States. The survey was performed by the National Center for Health Statistics (https://www.cdc.gov/nchs/nhanes/index.htm) and had a complex, multistage, stratified sampling design in order to provide nationally representative data. We conducted an analysis using the data collected from the adults aged over 60 years who participated in the NHANES 2011 to 2014 cycles. In the NHANES 2011–2012 survey, the unweighted response rate for participants aged 60–70, 70–80, and over 80 were 62.7%, 56.1%, and 46.4% respectively. In the NHANES 2013-2014 survey, the unweighted response rate for participants aged 60–70, 70–80, and over 80 were 62.9%, 59.8%, and 45.2% (https://wwwn.cdc.gov/nchs/nhanes/responserates.aspx). Participants without response to cognitive functioning tests (*n* = 286), without data of glucose metabolic components (*n* = 247), and those who were taking prescribed medications to treat obesity (*n* = 14) were excluded, yielding a primary analytical sample of 2,925, including 511 patients with diabetes who were using oral hypoglycemic agents or insulin (**Figure 1**). The survey collected data first through interviews at participants’ home by experienced interviewers using Computer-Assisted Personal Interviewing system, with a subsequent visit to a mobile examination center (MEC) where some clinical assessments and collection of biological specimens were carried out. The National Center for Health Statistics ethics review board reviewed and approved the survey and participants of NHANES gave informed consent prior to participation.

### Cognitive Functioning Assessment

Cognitive function was evaluated during the in-home interview using four separate tests that have been validated in American communities (15–17), including the Consortium to Establish a Registry for Alzheimer’s Disease Immediate Recall (CERAD-IR), the Consortium to Establish a Registry for Alzheimer’s Disease Delayed Recall (CERAD-DR), the AF, and the DSST.

CERAD-IR and CERAD-DR comprehensively measure both learning ability and memory (18). The CERAD-IR test consists of three consecutive learning trials with a maximum total score of 30. Participants were asked to read 10 unrelated words at the beginning and repeat the words as many as possible in each learning trial. The CERAD-DR occurred after the other cognitive tests (AF and DSST) were completed, in which the participants recalled the words presented in the CERAD-IR test. The AF evaluates categorical verbal fluency by counting the number of animals named by the participant in 1 min (19). The DSST is a widely used evaluation tool assessing processing speed, sustained attention, and working memory (20). The test was conducted using a paper form containing nine digit–symbol pairs. For this test, the participants had to fill in the blank boxes next to the digit with corresponding symbols within 2 min. In line with previous literature, we used the criteria of <17 for CERAD-IR (21), <5 for CERAD-DR (21), <14 for AF (22), and <34 for DSST (23) to identify potential cognitive impairment.

### Glucose Metabolic Components

HbA_1c_ measures were available for the entire sample and were conducted through a high-performance liquid chromatography method. Participants who were randomly assigned to the morning session were eligible for FPG and insulin measures, whereas only part of the morning session participants were suitable for a subsequent oral glucose tolerance test (OGTT). The exclusion criteria for OGTT included hemophilia or chemotherapy patients, fasting less than 9 h, refusing phlebotomy, failing to drink entire glucose solution, or currently receiving diabetes treatment. After the FPG test, participants were asked to drink a solution containing approximately 75 g of glucose and have a second venipuncture 2 h later. AIA-PACK IRI, a two-site immunoenzymometric assay, was performed to quantitively measure insulin in serum specimens; plasma glucose was measured using hexokinase assay. The homeostatic model assessment of insulin resistance (HOMA-IR) was calculated as [fasting glucose(mmol/L)] × [plasma insulin (μIU/ml)]/22.5 (24) and used as surrogate measurement of insulin resistance. Details of lab method, lab equipment, and lab site were described elsewhere (25, 26). According to the American Diabetes Association 2014 criteria (27), diabetes was defined as FPG ≥ 7.0 mmol/L, 2h-PG ≥ 11.1 mmol/L, or HbA_1c_ ≥ 6.5%. In the current study, the presence of diabetes was defined as FPG ≥ 7.0 mmol/L, 2h-PG ≥ 11.1 mmol/L, HbA_1c_ ≥ 6.5%, and receiving oral hypoglycemic agent treatment or insulin treatment. We calculated the number of glucose metabolic risk factors according to the presence of five glucose metabolic risk factors (1): FPG ≥ 7.0 mmol/L (2); HbA_1c_ ≥ 6.5% (3); HOMA-IR ≥ 4.11 (highest quartile) (4); receive hypoglycemic agent treatment; and (5) receive insulin treatment. The glucose metabolic health status was defined based on the number of glucose metabolic risk factors and thus classified into three categories: the healthiest glucose metabolism (without any risk factor), moderately healthy glucose metabolism (1–3 risk factors), and the least healthy glucose metabolism (4–5 risk factors).

### Covariates

Demographic statistics, sleep disorder, history of stroke, history of hyperlipidemia, smoking status, alcohol consumption, prescription medication, and mental health data were collected in the in-home interviews. In terms of age, responses of participants who were more than 80 years old were coded as “80” in order to protect participants’ privacy. Depressive symptoms were evaluated through Patient Health Questionnaire (PHQ-9), a nine-item depression screener that asked questions on the frequency of depression symptoms in the past 2 weeks. Each item was scored on a scale of 1–3 with a maximum total score of 27; higher scores represent more severe depression (28). Currently smoking was defined as having smoked at least 100 cigarettes in life, and current alcohol consumption was defined as having at least 12 alcohol drinks in the past year. Information on health status (history of stroke, history of hyperlipidemia, and sleep disorder) was collected by asking the questions “have you ever been told by a doctor or other health professional that you have stroke/hyperlipidemia/sleep disorder?”

Body and blood pressure (BP) measurements were taken at MEC by trained technicians. The BP examiners asked the participants to sit quietly for 5 min and subsequently obtained three consecutive systolic blood pressure (SBP) readings. Participants without suitable BP cuff, or with rashes, gauze dressings, casts, edema, paralysis, tubes, open sores or wounds, withered arms, a-v shunts, and radical mastectomy on both arms were excluded. Body mass index (BMI) was calculated as the weight in kilograms divided by height in meters squared. We applied BMI ≥ 30 kg/m^2^, a cutoff recommended for US adults (29), to identify obesity.

### Statistical Analysis

All statistical analyses were conducted using Stata version 11.0 and R version 4.0.3, accounting for the complex sampling design in NHANES. Each person in the survey was assigned a sample weight that was created by three steps (1): calculation of the base weight according to the possibility that his/her county, city block, household, and then herself/himself is selected (2); adjustment for nonresponse; and (3) post stratification adjustment to match the US Census population. Weights of the 2011–2012 cycle and 2013–2014 cycle were combined to create 4-year survey weights to adjust for unequal selection probability and non-response bias (30). Weights of the variable that were collected on the smallest number of respondents were recommended to be applied during analysis.

Weighted means with standard deviations for normally distributed data, medians with 25th–75th percentiles for skewed distributed variables, and percentages of categorical data were calculated. We performed *t*-tests for continuous data and design-based *χ* ^2^ tests for categorical data to determine statistical difference between characteristics of participants with normal and low scores in each cognitive test. Insulin and HOMA-IR were log-transformed before statistical comparisons owing to their nonnormal, positively skewed distributions. First, we used multivariable linear regression models to delineate the individual association of glucose metabolic components with cognitive function among participants without using oral hypoglycemic agents and insulin. To estimate whether the associations of individual glucose metabolic component with cognitive functions were modified by obesity status, we replicated linear regression analyses in both BMI stratification categories (<30 kg/m^2^ and ≥ 30 kg/m^2^). Design-based *t*-tests were used to determine difference between cognitive scores between patients using metformin and using other anti-diabetic medications. Linear regression analysis was performed to evaluate relationship between metformin use and cognitive function among 511 patients who were taking medications used to treat diabetes. Multiple logistic regression models and 95% confidence intervals (CIs) were used to estimate the odds of scoring low in different cognitive tests that are associated with the number of glucose metabolic risk components. Non-response to cognitive testing and measurement of glucose components for any reason was treated as missing data and not included in the analyses.

The covariates adjusted in the models are presented as follows: sex (male and female), race (Hispanic, White, Black, and Asian), education (<9th grade, 9–11th grade, high school, some college, and ≥college), sleep disorder (yes and no), history of stroke (yes and no), smoking (yes and no), alcohol consumption status (yes and no), history of hyperlipidemia (yes and no), BMI (continuous data), SBP (continuous data), and PHQ-9 scores (continuous data). Three models were generated for the analysis. Model 1 was univariate, and Model 2 was adjusted for age, sex, race, education level, PHQ-9 scores, sleep disorder, history of stroke, history of hyperlipidemia, BMI, and SBP. Model 3 was additionally adjusted for alcohol consumption and smoking. We selected Model 3 as the main adjustment model.

We used the software of Power Analysis & Sample Size 11.0 to conduct the power calculation (31). According to a previous published study that had evaluated the association between HbA_1c_ and cognitive assessment score (14), we calculated the statistical power of the present study by using the following parameters (1): a two-sided test at the 5% level (2); the null hypothesis of *β* of linear regression is 0 (3); the standard deviations of the HbA_1c_ in our study was 0.54 (4); an estimation of the correlation between HbA_1c_ and cognitive assessment score was 0.1 (5); the sample size is 2,414 (6); the *β* of linear regression between HbA_1c_ and CERAD-IR, CERAD-DR, DSST, and AF was −0.19, −0.51, −2.55, and −0.64 reported by the previous published study, respectively (14). The calculated statistical powers were all over 90%.

## Results

### Description of Population Characteristics

**Table 1** presents the characteristics of 2,925 participants. Participants scoring low in cognitive tests were significantly older and had higher SBP and higher 2h-PG than those with normal cognitive function. Differences were also observed in race and education constituent ratios between participants with normal and low cognitive scores. There was higher prevalence of impaired reaction on CERAD-IR and CERAD-DR among women compared with men, whereas gender difference was not significant regarding DSST and AF.

**Table 1:** Characteristics of participants with normal and low cognitive scores.

Characteristics	Total	CERAD-IR		CERAD-DR		DSST		AF	
		≥17 (*n* = 2016)	<17 (*n* = 863)	≥5 (*n* = 2102)	<5 (*n* = 769)	≥34 (*n* = 2074)	<34 (*n* = 710)	≥14 (*n* = 1975)	<14 (*n* = 885)
Age, years	69.42 ± 6.74	68.33 ± 6.06^*^	72.95 ± 7.74	68.30 ± 6.19**^#^**	73.11 ± 7.22	68.59 ± 6.05**^ǂ^**	73.13 ± 8.64	68.58 ± 6.13**^§^**	72.23 ± 7.97
Gender, %									
Male	45.4	73.0^*^	27.0	73.2**^#^**	26.8	85.0	15.0	78.8	21.2
Female	54.6	80.1	19.9	81.0	19.0	85.3	14.7	76.8	23.2
Race, %									
White	79.5	79.0	21.0	78.4	21.6	89.5	10.5	81.7	18.3
Black	8.2	71.5	28.5	72.8	27.2	64.2	35.8	57.4	42.6
Asian	3.4	73.1	26.9	83.0	17.0	84.3	15.7	54.3	45.7
Hispanic	7.3	62.7	37.3	69.6	30.4	57.3	42.7	67.2	32.8
Education, %									
<9th grade	6.2	43.5	56.5	56.7	43.3	33.5	66.5	49.6	50.4
9–11th grade	10.5	60.9	39.1	68.7	31.3	67.2	32.8	62.6	37.4
High school	21.8	74.0	26.0	74.3	25.7	83.4	16.6	71.2	28.8
Some college	31.3	82.0	18.0	82.5	17.5	91.0	9.0	82.3	17.7
≥ College	30.2	86.0	14.0	81.7	18.3	95.7	4.3	88.4	11.6
Oral hypoglycemic agents/insulin, %									
Yes	14.7	71.9	28.1	73.6	26.4	77.5	22.5	79.0**^§^**	21.0
No	85.3	77.7	22.3	78.1	21.9	86.5**^ǂ^**	13.6	70.1	29.9
Sleep disorder, %									
Yes	11.6	77.0	23.0	78.0	22.0	86.0ǂ	14.0	78.9^§^	21.1
No	88.4	77.0	23.0	77.5	22.5	85.2	14.9	77.6	22.4
History of stroke, %									
Yes	6.7	62.7^*^	37.3	66.2^#^	33.8	66.1^ǂ^	33.9	61.0**^§^**	39.0
No	93.3	77.9	22.1	78.2	21.8	86.4	13.6	79.0	21.0
History of hyperlipidemia, %									
Yes	57.5	77.4^*^	22.6	77.6^#^	22.5	85.7^ǂ^	14.3	78.0	22.0
No	42.5	76.7	23.3	77.8	22.2	85.2	14.8	77.7	22.3
Smoking									
Yes	50.3	77.5	22.5	78.7	21.3	84.8	15.2	78.0	22.0
No	49.7	76.2	23.8	76.2	23.8	85.5	14.5	77.4	22.6
Consuming alcohol, %									
Yes	72.4	79.9^*^	20.1	79.5^#^	20.5	88.4^ǂ^	11.6	81.1**^§^**	18.9
No	27.6	70.2	29.8	72.9	27.1	77.7	22.4	70.0	30.0
BMI, kg/m^2^	28.90 ± 6.24	29.18 ± 6.12^*^	28.02 ± 6.44	29.07 ± 6.15 **^#^**	28.37 ± 6.60	28.89 ± 5.78	28.81 ± 8.62	28.97 ± 5.82	28.70 ± 7.78
SBP, mmHg	131.68 ± 18.85	130.63 ± 17.34^*^	134.63 ± 23.30	130.76 ± 17.88**^#^**	134.53 ± 22.04	130.57 ± 16.73**^ǂ^**	137.28 ± 29.01	130.64 ± 17.49**^§^**	135.23 ± 23.09
PHQ-9 score	2.93	2.83 ± 3.84^*^	3.43 ± 5.37	2.83 ± 3.90**^#^**	3.42 ± 5.22	2.66 ± 3.60**^ǂ^**	4.46 ± 7.22	2.74 ± 3.68**^§^**	3.75 ± 5.98
HOMA-IR	2.41 (1.47–4.11)	2.43 (1.43–4.05)	2.39 (1.52–4.36)	2.39 (1.42–4.05)	2.48 (1.55–4.34)	2.43 (1.47–4.04)	2.50 (1.48–4.5)	2.42 (1.48–4.11)	2.36 (1.36–4.11)
FPG, mmol/L	5.99 ± 1.71	5.97 ± 1.59	6.05 ± 2.11	5.96 ± 1.62	6.09 ± 2.02	5.96 ± 1.58	6.13 ± 2.22	5.96 ± 1.49	6.06 ± 2.29
Insulin, μIU/ml	9.41 (6.06–15.13)	9.40 (6.01–15.04)	9.59 (6.28–15.27)	9.32 (6.04–14.97)	9.89 (6.41–15.56)	9.41 (6.06–15.07)	9.87 (6.03–15.54)	9.42 (6.13–15.07)	9.16 (5.88–15.07)
HbA_1c_, %^1^	5.90 ± 0.91	5.88 ± 0.85	5.97 ± 1.06	5.88 ± 0.88 **^#^**	5.98 ± 0.99	5.85 ± 0.78**^ǂ^**	6.15 ± 1.60	5.87 ± 0.81**^§^**	6.03 ± 1.23
2h-PG, mmol/L	7.47 ± 2.98	7.36 ± 2.85^*^	8.01 ± 3.38	7.31 ± 2.90 **^#^**	8.21 ± 3.17	7.25 ± 2.65**^ǂ^**	8.74 ± 4.71	7.36 ± 2.82**^§^**	7.95 ± 3.59

*CERAD-IR ≥ 17 vs. CERAD-IR < 17, p < 0.05; ^#^CERAD-DR ≥ 5 vs. CERAD-DR < 5, p < 0.05; ^ǂ^DSST ≥ 34 vs. DSST < 34, p < 0.05; ^§^AF ≥ 14 vs. AF < 14, p < 0.05. Constituent ratios of race and education differed significantly between participants with normal and low scores in all cognitive tests according to design-based χ^2^ tests.

Data were presented as mean ± standard deviations for normal distribution variables, median (25th–75th percentile) for skewed distribution variables, and weighted percentages for categorical variables.

Design-based t tests for continuous data and design-based χ^2^ tests for categorical data were performed to determine statistical difference between characteristics of participants with normal and low cognitive scores. Skewed distribution variables (HOMA-IR, Insulin) were log-transformed before statistical comparisons.

AF, Animal Fluency Test; BMI, body mass index; CERAD-IR, Consortium to Establish a Registry for Alzheimer’s Disease Immediate Recall; CERAD-DR, Consortium to Establish a Registry for Alzheimer’s Disease Delayed Recall; DSST, Digit Symbol Substitution Test; HOMA-IR, homoeostasis model assessment of insulin resistance; FPG, fasting plasma glucose; HbA_1c_, glycated hemoglobin; 2h-PG, 2-h postload glucose; PHQ-9, Patient Health Questionnaire (a nine-item screening instrument that asked questions about the frequency of symptoms of depression over the past 2 weeks); SBP, systolic blood pressure.

**Figure 2:**
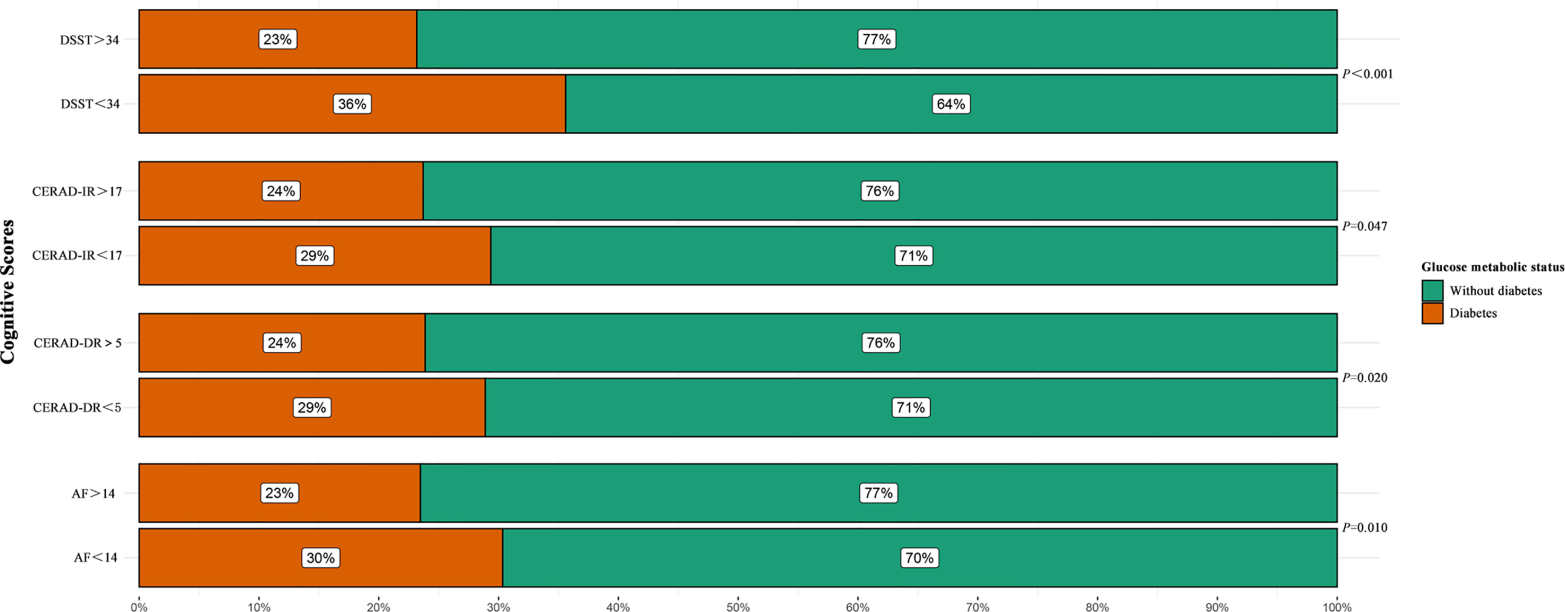
Percentages of patients with diabetes among participants with normal and low scores in each cognitive test. Design-based *χ* ^2^ test was performed to determine the difference between the constituent ratios of diabetes among participants with normal and low scores in each cognitive test. AF, Animal Fluency Test; CERAD-IR, Consortium to Establish a Registry for Alzheimer’s Disease Immediate Recall; CERAD-DR, Consortium to Establish a Registry for Alzheimer’s Disease Delayed Recall; DSST, Digit Symbol Substitution Test.

As shown in **Figure 2**, diabetes patients accounted for about 30% of participants who scored low in CERAD-IR, CERAD-DR, and AF. It is notable that more than 36% of individuals who scored low in DSST were patients with diabetes. By contrast, diabetes patients only accounted for less than a quarter of those with normal cognitive scores. The differences between participants with normal and low cognitive scores were statically significant according to design-based *χ* ^2^ tests (*p* = 0.047 for CERAD-IR; *p* = 0.020 for CERAD-DR; *p* = 0.010 for AF; *p* < 0.001 for DSST).

### Individual Association of Glucose Metabolic Components With Cognitive Function

**Table 2** shows the coefficients of the relationships of individual glucose metabolic component with cognitive scores. We found significant associations of 2h-PG with scores of CERAD-IR, CERAD-DR, and DSST in Model 1, but such associations were only maintained for CERAD-DR (*β* = −0.070, 95% CI −0.134 to −0.005) in the fully adjusted model. HbA_1c_ was inversely associated with scores in all four cognitive tests in the unadjusted model, and the association remained robust even after adjustment for all the confounding factors for CERAD-IR (*β* = −0.319, 95% CI −0.585 to −0.053), CERAD-DR (*β* = −0.227, 95% CI −0.405 to −0.050), and DSST (*β* = −1.914, 95% CI −3.537 to −0.291), but not for AF. The significant negative association of CERAD-IR with HOMA-IR (*β* = −0.830, 95% CI −1.655 to −0.005) and insulin (*β* = −1.013, 95% CI −1.969 to −0.058) was detected after adjustment of covariates. We did not observe any association between FPG and cognitive function.

**Table 2:** Individual association of glucose metabolic components with cognitive function.

	CERAD-IR		CERAD-DR		DSST		AF	
	*β*	95% CI	*β*	95% CI	*β*	95% CI	*β*	95% CI
**HOMA-IR**								
Model 1	−0.720	−1.618 to 0.178	−0.204	−0.634 to 0.226	−1.934	−5.584 to 1.716	0.049	−1.115 to 1.214
Model 2	−0.846	−1.676 to −0.017	−0.281	−0.748 to 0.186	−2.024	−5.741 to 1.693	−0.243	−1.333 to 0.847
Model 3	−0.830	−1.655 to −0.005	−0.279	−0.729 to 0.171	−1.814	−5.340 to 1.711	−0.193	−1.248 to 0.861
**Insulin**								
Model 1	−0.860	−1.912 to 0.192	−0.260	−0.780 to 0.261	−2.226	−6.274 to 1.821	−0.035	−1.281 to 1.211
Model 2	−1.035	−1.997 to −0.073	−0.377	−0.928 to 0.175	−2.641	−6.932 to 1.650	−0.343	−1.490 to 0.805
Model 3	−1.013	−1.969 to −0.058	−0.374	−0.904 to 0.157	−2.328	−6.422 to 1.766	−0.287	−1.390 to 0.817
**FPG**								
Model 1	−0.034	−0.192 to 0.123	0.014	−0.072 to 0.100	−0.179	−0.974 to 0.615	0.028	−0.214 to 0.270
Model 2	−0.017	−0.149 to 0.115	0.031	−0.037 to 0.099	0.034	−0.520 to 0.587	−0.028	−0.291 to 0.236
Model 3	−0.019	−0.156 to 0.118	0.030	−0.038 to 0.098	−0.005	−0.544 to 0.535	−0.026	−0.293 to 0.241
**HbA_1c_**								
Model 1	−0.820	−1.169 to −0.471	−0.428	−0.623 to −0.234	−4.472	−6.524 to −2.421	−0.897	−1.680 to −0.114
Model 2	−0.305	−0.567 to −0.042	−0.222	−0.395 to −0.049	−1.882	−3.567 to −0.198	−0.245	−1.000 to 0.510
Model 3	−0.319	−0.585 to −0.053	−0.227	−0.405 to −0.050	−1.914	−3.537 to −0.291	−0.205	−0.967 to 0.557
**2h-PG**								
Model 1	−0.258	−0.368 to −0.147	−0.138	−0.197 to −0.080	−1.044	−1.673 to −0.415	−0.176	−0.373 to 0.021
Model 2	−0.099	−0.214 to 0.016	−0.071	−0.134 to −0.008	−0.266	−0.670 to 0.139	0.013	−0.146 to 0.173
Model 3	−0.094	−0.211 to 0.023	−0.070	−0.134 to −0.005	−0.232	−0.634 to 0.169	0.025	−0.133 to 0.183

Participants with administration of oral hypoglycemic agents and insulin (*n* = 511) were excluded from the analysis.

Insulin and HOMA-IR were log-transformed before analysis; estimates (*β*) for insulin and HOMA-IR were reported per one log base 10 increase.

Model 1 was univariate; Model 2 was adjusted for age, gender, race, education, sleep disorder, history of stroke, history of hyperlipidemia, BMI, and SBP. Model 3 was Model 2 + smoking and alcohol consumption.

SE, standard error; CI, confidence interval; CERAD-IR, Consortium to Establish a Registry for Alzheimer’s Disease Immediate Recall; CERAD-DR, Consortium to Establish a Registry for Alzheimer’s Disease Delayed Recall; DSST, Digit Symbol Substitution Test; AF, Animal Fluency Test; HOMA-IR, homoeostasis model assessment of insulin resistance; FPG, fasting plasma glucose; HbA_1c_, glycated hemoglobin; 2h-PG, 2-hour postload glucose; SE, standard error.

### Individual Association of Glucose Metabolic Components With Cognitive Function Stratified by Obesity Status

As shown in **Table 3**, in the BMI stratified analysis, we found more prominent associations of individual glucose metabolic component with cognitive functions among adults without obesity (BMI < 30 kg/m^2^) after adjustment for age, gender, race, education, sleep disorder, history of stroke, history of hyperlipidemia, BMI, SBP, smoking, and alcohol consumption. Among participants with BMI < 30 kg/m^2^, HbA_1c_ and HOMA-IR were negatively associated with CERAD-IR (HOMA-IR: *β* = −1.778, 95% CI −2.842 to −0.714; HbA_1c_: *β* = −0.469, 95% CI −0.907 to −0.031) and CERAD-DR (HOMA-IR: *β* = −0.842, 95% CI −1.399 to −0.284; HbA_1c_: *β* = −0.310, 95% CI −0.572 to −0.049). Insulin was reversely associated with cognitive function based on CERAD-IR (*β* = −2.053, 95% CI −3.283 to −0.822) and CERAD-DR (*β* = −1.034, 95% CI −1.680 to −0.387). It is notable that no significant association was found between FPG and any cognitive test in both BMI categories.

**Table 3:** Individual association of glucose metabolic components with cognitive function stratified by obesity status.

	CERAD-IR		CERAD-DR		DSST		AF	
	*β*	95% CI	*β*	95% CI	*β*	95% CI	*β*	95% CI
**HOMA-IR**								
BMI < 30 kg/m^2^	−1.778	−2.842 to −0.714	−0.842	−1.399 to −0.284	−3.214	−7.073 to 0.645	−0.771	−2.089 to 0.548
BMI ≥ 30 kg/m^2^	0.880	−0.258 to 2.018	0.628	−0.085 to 1.341	1.786	−3.470 to 7.042	0.934	−0.888 to 2.755
**Insulin**								
BMI < 30 kg/m^2^	−2.053	−3.283 to −0.822	−1.034	−1.680 to −0.387	−4.210	−8.491 to 0.072	− 0.889	−2.312 to 0.535
BMI ≥ 30 kg/m^2^	0.949	−0.332 to 2.229	0.753	−0.050 to 1.555	2.654	−3.449 to 8.756	0.969	−1.103 to 3.042
**FPG**								
BMI < 30 kg/m^2^	−0.115	−0.260 to 0.030	0.014	−0.061 to 0.089	0.121	−0.583 to 0.825	−0.099	−0.354 to 0.156
BMI ≥ 30 kg/m^2^	0.145	−0.108 to 0.397	0.042	−0.127 to 0.212	−0.317	−1.307 to 0.672	0.104	−0.242 to 0.450
**HbA_1c_**								
BMI < 30 kg/m^2^	−0.469	−0.907 to −0.031	−0.310	−0.572 to −0.049	−1.738	−3.372 to 0.105	−0.344	−1.056 to 0.368
BMI ≥ 30 kg/m^2^	−0.065	−0.719 to 0.588	−0.072	−0.413 to 0.270	−2.314	−5.373 to 0.746	0.058	−1.291 to 1.407
**2h-PG**								
BMI < 30 kg/m^2^	−0.127	−0.283 to 0.029	−0.072	−0.155 to 0.010	−0.166	−0.740 to 0.408	0.058	−0.095 to 0.212
BMI ≥ 30 kg/m^2^	−0.079	−0.249 to 0.091	−0.089	−0.188 to 0.010	−0.425	−0.906 to 0.057	−0.027	−0.218 to 0.164

Participants with administration of oral hypoglycemic agents and insulin (n = 511) were excluded from the analysis.

Insulin and HOMA-IR were log-transformed before analysis; estimates (β) for insulin and HOMA-IR were reported per one log base 10 increase.

Linear regression models were adjusted for age, gender, race, education, sleep disorder, history of stroke, history of hyperlipidemia, BMI, SBP, smoking, and alcohol consumption.

SE, standard error; CI, confidence interval; CERAD-IR, Consortium to Establish a Registry for Alzheimer’s Disease Immediate Recall; CERAD-DR, Consortium to Establish a Registry for Alzheimer’s Disease Delayed Recall; DSST, Digit Symbol Substitution Test; AF, Animal Fluency Test; HOMA-IR, homoeostasis model assessment of insulin resistance; FPG, fasting plasma glucose; HbA_1c_, glycated hemoglobin; 2h-PG, 2-h postload glucose; BMI, body mass index.

### Combined Associations of Glucose Metabolic Components With Cognitive Function

**Figure 3:**
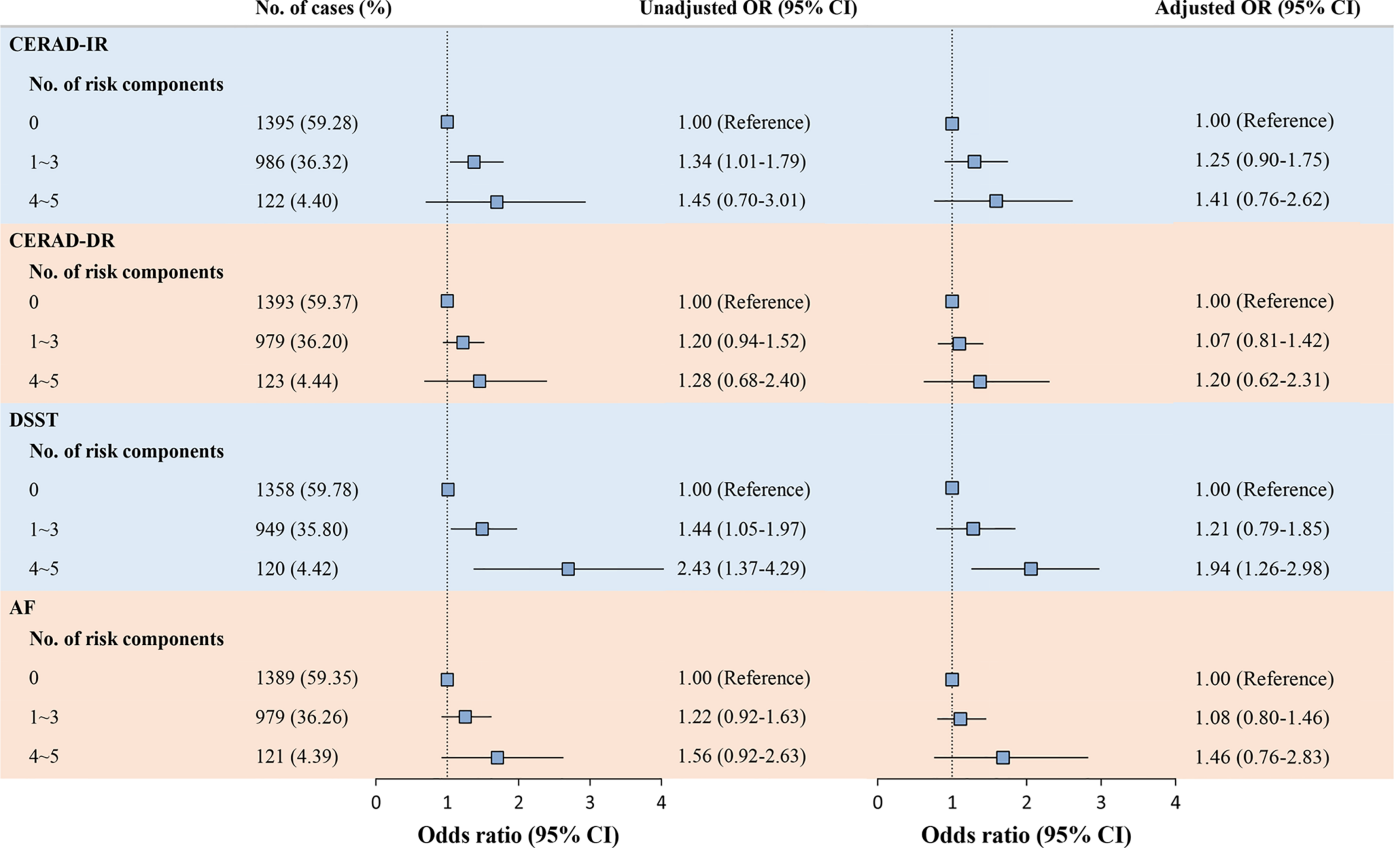
Associations of the number of glucose metabolic risk components with cognitive function. Participants with missing data on any of the glucose components (*n* = 387) were excluded from the analysis. Logistic regression models were used to generate odds ratios (ORs) and corresponding 95% confidence intervals (95% CIs) to detect the odds of cognitive impairment, adjusted for age, gender, race, education, sleep disorder, history of stroke, history of hyperlipidemia, BMI, SBP, smoking, and alcohol consumption. AF, Animal Fluency Test; BMI, body mass index; CERAD-IR, Consortium to Establish a Registry for Alzheimer’s Disease Immediate Recall; CERAD-DR, Consortium to Establish a Registry for Alzheimer’s Disease Delayed Recall; DSST, Digit Symbol Substitution Test; SBP, systolic blood pressure.

After adjusting for covariates, ORs of cognitive impairment based on CERAD-IR, CERAD-DR, and AF tests increased with the number of glucose metabolic risk components but did not reach statistical significance. Importantly, participants with least healthy glucose metabolic status (four to five risk components) have nearly doubled OR of scoring low in DSST compared with those with the healthiest glucose metabolic status (OR 1.94, 95% CI 1.26 to 2.98; **Figure 3**).

### Associations of Metformin Use With Cognitive Function

**Figure 4:**
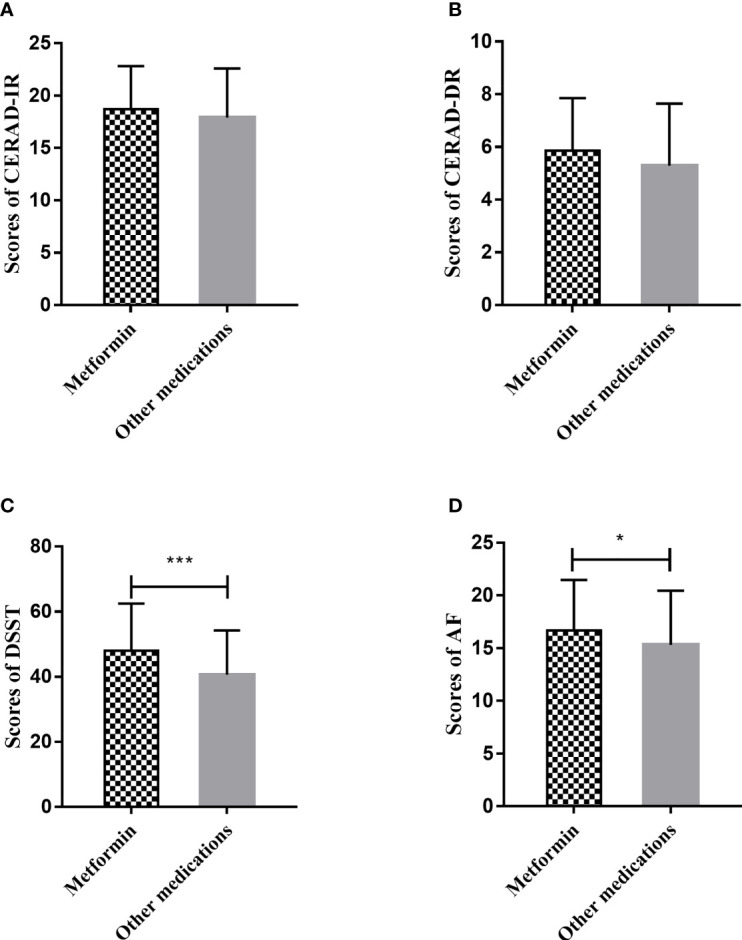
Cognitive scores of diabetes patients according to whether they are using metformin. **(A)** Comparison of CERAD-IR scores of patients using metformin and that of patients using other anti-diabetic medications. **(B)** Comparison of CERAD-DR scores of patients using metformin and that of patients using other anti-diabetic medications. **(C) **Comparison of DSST scores of patients using metformin and that of patients using other anti-diabetic medications. **(D)** Comparison of AF scores of patients using metformin and that of patients using other anti-diabetic medications. Design-based *t*-test was performed to determine the difference between cognitive scores of diabetes patients taking metformin and those taking other anti-diabetic medications. *p < 0.05; ***p < 0.001. AF, Animal Fluency Test; CERAD-IR, Consortium to Establish a Registry for Alzheimer’s Disease Immediate Recall; CERAD-DR, Consortium to Establish a Registry for Alzheimer’s Disease Delayed Recall; DSST, Digit Symbol Substitution Test.

Among 511 participants who were taking oral hypoglycemic agents or using insulin to treat diabetes, 311 were using metformin. As shown in **Figure 4**, patients using metformin scored significantly higher in DSST and AF, compared with patients who were using other anti-diabetic medications. In the linear regression analysis (**Table 4**), we found significant associations of metformin use with better language fluency, as evaluated with AF (*β* = 1.362, 95% CI 0.270 to 2.454), and better executive function, as evaluated by DSST (*β* = 7.406, 95% CI 4.373 to 10.440). After adjusting for aforementioned covariates, metformin use remained significantly associated with better executive function based on DSST (*β* = 4.184, 95% CI 1.655 to 6.713).

**Table 4:** Associations between metformin use and cognitive function.

	*β*	95% CI	*p*
**CERAD-IR**			
Model 1	0.776	−0.321 to 1.874	0.159
Model 2	0.126	−0.847 to 1.100	0.793
Model 3	0.027	−1.060 to 1.113	0.960
**CERAD-DR**			
Model 1	0.568	−0.122 to 1.257	0.103
Model 2	0.347	−0.299 to 0.993	0.282
Model 3	0.353	−0.324 to 1.031	0.295
**DSST**			
Model 1	7.406	4.373 to 10.440	<0.001
Model 2	4.745	1.942 to 7.547	0.002
Model 3	4.184	1.655 to 6.713	0.002
**AF**			
Model 1	1.362	0.270 to 2.454	0.016
Model 2	0.341	−0.613 to 1.296	0.471
Model 3	0.194	−0.904 to 1.293	0.721

Participants with administration of oral hypoglycemic agents and insulin (n = 511) were included in the analysis.

Model 1 was univariate; Model 2 was adjusted for age, gender, race, education, sleep disorder, history of stroke, history of hyperlipidemia, BMI and SBP. Model 3 was Model 2 + smoking and alcohol consumption.

SE, standard error; CI, confidence interval; CERAD-IR, Consortium to Establish a Registry for Alzheimer’s Disease Immediate Recall; CERAD-DR, Consortium to Establish a Registry for Alzheimer’s Disease Delayed Recall; DSST, Digit Symbol Substitution Test; AF, Animal Fluency Test.

## Discussion

To our knowledge, our study is the first to examine the individual and combined associations of glucose metabolic components with domain-specific cognitive function comprehensively among US older adults. Our study demonstrated that severe insulin resistance estimated using HOMA-IR and insulin concentration and high glycemic level estimated using HbA_1c_ and 2h-PG were negatively associated with cognitive function in key domains except for verbal fluency, especially among participants without obesity. Patients with four to five glucose metabolic risk components had significantly worse performance in DSST, indicating poorer processing speed, sustained attention, and working memory in relation to worse glucose metabolism. Furthermore, metformin use was associated with executive function as evaluated by DSST among diabetes patients.

The association of HbA_1c_ and cognitive impairment is still debated. Elevated HbA_1c_ level has been shown to be related to increased odds of dementia in epidemiological studies (32), though not all studies found such associations (33). A recent study (14) carried out in the NHANES 2011–2014 population did find cognitive impairment in relation to elevated HbA_1c_. Our study, however, extended the knowledge by comprehensively taking multiple glycemic and insulin resistance indicators, obesity status, and anti-diabetic medications into account.

Studies examining other glucose metabolic components, such as FPG and 2h-PG, in relation to cognitive outcomes are more limited. Most studies reported no relation between FPG concentration and cognition (5), consistent with findings of our study. The level of 2h-PG indicates moderate to severe insulin resistance and impaired late-phase insulin secretory response to OGTT; thus, it is different from FPG with regard to pathophysiology and odds of diabetes-related clinical outcomes (34). The Hisayama Study (35) reported increased odds of all-cause dementia, Alzheimer’s disease, and vascular dementia in relation to elevated 2h-PG, rather than FPG. Given the fact that memory loss is the key symptom of Alzheimer’s disease (36), our study, which showed 2h-PG negatively associated with memory, supports results derived from the Hisayama Study.

Insulin resistance, a state of inadequate response to insulin, is considered to be a hallmark of type 2 diabetes, and it is also observed in neurodegenerative disease (37). In addition, hyperinsulinemia is a sign of insulin resistance (38). It has been documented that both hyperinsulinemia and insulin resistance are related to poorer cognitive performance and dementia among a population without diabetes (39). Regarding diabetes patients, a cross-sectional study derived from the CAROLINA trial showed no significant association of insulin resistance with cognitive outcomes (40). Some cross-sectional studies reported significant, but weak correlation between fasting insulin concentration and Mini Mental State Examination score (40, 41) or memory (42).

Over the past decade, the prevalence of obesity in the United States has increased substantially and steadily (43). Results from different studies investigating association of obesity with dementia have been conflicting. It has been documented that mid-life obesity is related to poorer cognitive function in late-life (44, 45). Nevertheless, regarding elder adults, several reports showed impaired cognitive function in relation to increased BMI in late life (46), but more studies indicated opposite results (47, 48). In the current study, we observed stronger and more pronounced associations of cognitive functions with HOMA-IR, insulin, and HbA_1c_ among adults with BMI < 30 kg/m^2^ compared with those with obesity.

We conducted risk stratification of cognitive impairment based on glucose metabolic health status. Relative to the group without any risk component, the odds of poor performance in DSST increased significantly with the number of metabolic risk components. DSST evaluates important aspects of executive function, including processing speed, sustained attention, and working memory (20). Several studies have reported consistent results that cognitive impairments were more prominent in the executive domain among patients with type 2 diabetes (49, 50); this might be in part attributed to decline in motor function due to peripheral neuropathy of diabetes patients (51).

Metformin, recommended globally as first-line treatment for type 2 diabetes, could reduce advanced glycation end products (52), which promote tissue degeneration and the microvascular complications of hyperglycemia. Epidemiological studies also support the beneficial associations of metformin with cognitive function (9, 10, 53). In support, we observed better processing speed associated with metformin use among diabetes patients who were using oral hypoglycemic agents or insulin.

Glucose metabolic disorders and cognitive impairment frequently coexist because they shared pathological mechanisms. Endothelial dysfunction related to hyperglycemia increased the accumulation of toxic lipids and advanced glycation end products, leading to reduced cerebral blood flow (54). Furthermore, insulin could protect neurons from Aβ synaptotoxicity and modulates Aβ clearance through its effects on lipid metabolism and proteases, such as the insulin-degrading enzyme (39). However, peripheral insulin resistance, and subsequent chronic hyperinsulinemia, could reduce the transport of insulin through the brain–blood barrier (55), which was in line with our findings that elevated fasting insulin and insulin resistance were associated with worse cognition.

The primary strengths of this study include a nationally representative sample, comprehensive measurements of cognitive functions in multiple domains, and glucose metabolic components. Our study does have a number of limitations. Firstly, owing to the cross-sectional design of our data, a causal relation cannot be established between glucose metabolic components and cognitive functions. Furthermore, we lacked parameters evaluating chronic inflammation, which was, however, identified as a potential risk factor for cognitive dysfunction in people with diabetes (56). Finally, because only participants assigned to the morning session were available for measurements of FPG and insulin, and an even smaller part of individuals in the morning session were suitable for the oral glucose tolerance test, our sample size was limited when doing most evaluations.

## Conclusions

The nationwide cross-sectional study showed that both insulin resistance and glycemic level were reversely related to cognitive function in multiple domains, including learning, memory, and executive functions, but not in language fluency. In addition, such associations seem to be more prominent among adults without obesity. Our findings add to the existing evidence supporting the beneficial associations of metformin with cognition and also underline the crosstalk between glycemic metabolism and cognitive functions in key domains, especially among older adults without obesity.

## Data Availability Statement

The datasets presented in this study can be found in online repositories. The names of the repository/repositories and accession number(s) can be found at: https://www.cdc.gov/nchs/nhanes/index.htm.

## Author Contributions

GN, YL, and ML conceived and designed the study. RH, YX, RZ, JLi, and ML analyzed data. YX, YB, WW, ML, YL, and GN interpreted the data. RH and RZ drafted the manuscript. QC, TH, ZZ, JLi, and MX revised it. YC, JLu, and TW collected data. All authors agreed to be accountable for all aspects of the work and approved the final version of the paper. ML, YL, and GN are the guarantors of this work and, as such, had full access to all the data in the study and take responsibility for the integrity of the data and the accuracy of the data analysis.

## Funding

This work was supported by grants from the Shanghai Shenkang Hospital Development Center (SHDC12019101).

## Conflict of Interest

The authors declare that the research was conducted in the absence of any commercial or financial relationships that could be construed as a potential conflict of interest.

## Publisher’s Note

All claims expressed in this article are solely those of the authors and do not necessarily represent those of their affiliated organizations, or those of the publisher, the editors and the reviewers. Any product that may be evaluated in this article, or claim that may be made by its manufacturer, is not guaranteed or endorsed by the publisher.
